# Epidemic changepoint detection in the presence of nuisance changes

**DOI:** 10.1007/s00362-022-01307-x

**Published:** 2022-04-04

**Authors:** Julius Juodakis, Stephen Marsland

**Affiliations:** School of Mathematics and Statistics, Victoria University of Wellington, PO Box 600, Wellington, 6140 New Zealand

**Keywords:** Changepoint detection, Piecewise stationary time series, Segmentation, Stochastic gradient methods

## Abstract

Many time series problems feature epidemic changes—segments where a parameter deviates from a background baseline. Detection of such changepoints can be improved by accounting for the epidemic structure, but this is currently difficult if the background level is unknown. Furthermore, in practical data the background often undergoes nuisance changes, which interfere with standard estimation techniques and appear as false alarms. To solve these issues, we develop a new, efficient approach to simultaneously detect epidemic changes and estimate unknown, but fixed, background level, based on a penalised cost. Using it, we build a two-level detector that models and separates nuisance and signal changes. The analytic and computational properties of the proposed methods are established, including consistency and convergence. We demonstrate via simulations that our two-level detector provides accurate estimation of changepoints under a nuisance process, while other state-of-the-art detectors fail. In real-world genomic and demographic datasets, the proposed method identified and localised target events while separating out seasonal variations and experimental artefacts.

**Supplementary Information:**

The online version contains supplementary material available at 10.1007/s00362-022-01307-x.

## Introduction

The problem of identifying when the probability distribution of a time series changes—changepoint detection—has been studied since the middle of the 20th century. Early developments stemmed from operations research (Page 1954). However, as automatic and continuous data collection became more common, many new use cases for changepoint detection have arisen, such as seismic events (Li et al 2016), epidemic outbreaks (Texier et al 2016), gravity wave search (McNabb et al 2004), and network traffic spikes (Hochenbaum et al 2017). Stimulated by such practical interest, the growth of corresponding statistical theory has been rapid, as reviewed in Aminikhanghahi and Cook (2016), Niu et al (2016) and Truong et al (2020).

Different applications pose different statistical challenges. If a single drastic change may be expected, such as when detecting machine failure, the goal is to find a method with minimal time to detection and a controlled false alarm rate (Lau and Tay 2019). More often, both the number and locations of changepoints must be estimated; the challenge then is to achieve this in a computationally efficient way. Some problems, such as peak detection in sound (Mesaros et al 2017) or genetic data (Hocking et al 2017), feature epidemic segments— changepoints followed by a return to the background level—and incorporating this constraint can improve detection or simplify post-processing of outputs.

Current detection methods that do incorporate a background level assume it to be stable throughout the data (e.g., Fisch et al 2018; Zhao and Yau 2019). However, this is not realistic in common applications. In genetic analyses such as measurements of protein binding along DNA there may be large regions where the background level is shifted due to structural variation in the genome or technical artefacts (Zhang et al 2008). Similarly, a standard task in sound processing is to detect speech in the presence of dynamic background chatter (Mesaros et al 2017). In various datasets from epidemiology or climatology, such as wave height measurements (Killick et al 2012), seasonal effects are observed as recurring background changes and will interfere with the detection of shorter events. Methods that assume a constant background will be inaccurate in these cases, while ignoring the epidemic structure entirely would cost detection power and complicate the interpretation of outputs.

Our goal is to develop a general method for detecting epidemic changepoints in the presence of nuisance changes in the background. Furthermore, we allow the two types of changes to affect the same parameter, and assume that they are only distinguished by their duration: this would allow analysis of the examples above, which share the property that the nuisance process is slower. The closest research to ours is that of Lau and Tay (2019) for detecting failure of a machine that can be switched on, and thus undergo an irrelevant change in the background level. However, the setting there concerned a single change with known background and nuisance levels; in contrast, we are motivated by the case where multiple changes may be present, with only duration distinguishing their types. Detection then requires two novel developments: (1) rapid estimation of local background level, (2) modelling and distinguishing the two types of potentially overlapping segments.

These developments are presented in this paper as follows: after a background section we present a new algorithm that simultaneously detects epidemic changepoints and estimates the unknown background level (Sect. 3). The convergence and consistency of this algorithm are proved. While this algorithm is of its own interest, we use it to build a detector that allows local variations in the background, i.e., nuisance changes, in Sect. 4. In Sect. 5 we investigate the algorithms using simulations, before showing how the proposed nuisance-robust detector can be applied to two problems: detecting histone modifications in human genome, while ignoring structural variations, and detecting the effects of the COVID-19 pandemic in mortality data, robustly to seasonal effects. Compared to state-of-the-art methods, the proposed detector produced lower false-alarm rates (or more parsimonious models), while retaining accurate detection of true signal peaks.

## Background

In the general changepoint detection setup, the data is a sequence $x_{0:n} = \{x_0, \ldots , x_n\}$, split by changepoints $0<\tau _1<\tau _2<\cdots \tau _k<n$ into $k+1$ segments. The observations within each segment are drawn from a distribution $f(x; \theta )$, with potentially different values of parameter $\theta$ for each segment. The most common example is the change in mean of a Gaussian, i.e., for each $t \in [\tau _i, \tau _{i+1})$, $x_t \sim {\mathcal {N}}(\mu _i, \sigma ^2)$, for known, fixed $\sigma ^2$. (We assume $\theta \in {\mathbb {R}}^1$ to keep notation clearer, but multidimensional problems are also common.)

The aim is to estimate the number and position of all changepoints $\{ \tau _i \}$ in the data. A common approach is to use a penalised likelihood cost: define a segment cost function $C(x_{a:b}; \theta ) = -\log f(x_{a:b}; \theta )$, and a penalty *p*(*k*) for the number of changepoints *k*. The full cost of $x_{\tau _0+1:\tau _{k+1}}$ (where $\tau _0 = -1$ and $\tau _{k+1} = n$) with segment parameters $\varvec{\theta }$ then is (here and further vectors are denoted in bold):

1 $$\begin{aligned} F(n; \varvec{\tau }, \varvec{\theta }, k) = \sum _{i=0}^{k} C(x_{\tau _i+1:\tau _{i+1}}; \theta _i) + p(k). \end{aligned}$$

Changepoint number and positions are estimated by finding $F(n) = \min F(n; \varvec{\tau }, \varvec{\theta }, k)$. Such estimation has been shown to be consistent for a range of different data generation models (Fisch et al 2018; Zheng et al 2019).

For this problem, computing the true minimum is hard—a naïve brute force approach would require ${\mathcal {O}}(2^n)$ tests. Approaches to reducing this fall into two broad classes: (1) simplifying the search by memoisation and pruning of paths (Jackson et al 2005; Killick et al 2012; 2) using greedy methods to find approximate solutions faster (Fryzlewicz 2014; Baranowski et al 2019). In both classes, there are methods that can consistently estimate changepoints in linear time under certain conditions.

The first category is more relevant here. It is based on the Optimal Partitioning (OP) algorithm (Jackson et al 2005). Let the data be partitioned into discrete blocks $B_i: \bigcup _i B_i = x_{0:n}$, so $B_i \cap B_j = \emptyset , \forall i \ne j$. A function *V* that maps each set of blocks $P_j=\{B_i\}$ to a cost is block-additive if:

2 $$\begin{aligned} \forall P_1, P_2, V(P_1 \cup P_2) = V(P_1) + V(P_2). \end{aligned}$$

If each segment incurs a fixed penalty $\beta = p(k)/k$, then the cost *F* defined in (1) is block-additive over segments, and can be defined recursively:

$$\begin{aligned} F(s) = \min _t \left( F(t) + C(x_{t+1:s}) + \beta \right) . \end{aligned}$$

In OP, this cost is calculated for each $s \le n$, and thus its minimisation requires ${\mathcal {O}}(n^2)$ evaluations of *C*. Furthermore, when the cost function *C* is such that for all $a \le b < c$:

3 $$\begin{aligned} C(x_{a:c}) \ge C(x_{a:b}) + C(x_{b+1:c}) \end{aligned}$$

then at each *s* it can be determined that some candidate segmentations cannot be “rescued” by further segments, and so they can be pruned from the search space. This approach further reduces the complexity, and gave rise to the family of algorithms called PELT, Pruned Exact Linear Time (Killick et al 2012). Note that OP and PELT do not rely on any probabilistic assumptions and find the exact minimum. Other pruning schemes are available as well, with different constraints (Maidstone et al 2016).

In this paper, we focus on the *epidemic* variation of the basic changepoint model—here, a change in regime appears for a finite time and then the process returns to the background level. The pairs of changepoints $s_i, e_i$ define segments, outside of which the data is drawn from a background distribution $f_B$. The data model then becomes:

4 $$\begin{aligned} f(x_t) = {\left\{ \begin{array}{ll} f_S(x_t; \theta _i) &{} \text { if } \exists i: s_i \le t \le e_i \\ f_B(x_t) &{} \text { otherwise}. \end{array}\right. } \end{aligned}$$

Various methods for multiple changepoint detection in this setting have been proposed (Jeng et al 2010; Fisch et al 2018; Zhao and Yau 2019). These use a cost function that includes a separate term $C^0$ for the background points:

5 $$\begin{aligned} F(n; \{(s_i, e_i)\}_k, \varvec{\theta }, k) = \sum _{i=1}^k C(x_{s_i:e_i}; \theta _i) + C^0(\{x_t : t \notin \bigcup _i[s_i,e_i]\}; \theta _0) + p(k) \end{aligned}$$

A common choice for the background distribution is some particular “null” case of the segment distribution family, so that $f_B(x) = f_S(x; \theta _0)$ and $C^0(\cdot ) = -\log f_B(\cdot ) = C(\cdot ; \theta _0)$. However, while the value of $\theta _0$ is known in some settings (such as two copies of DNA in genetic data), often it needs to be estimated. Since $\theta _0$ is shared across all background points, the cost function is no longer block-additive as in (2), and OP and PELT algorithms cannot be directly applied.

One solution is to substitute the unknown parameter with some robust estimate of it, based on the unsegmented series $x_{0:n}$. The success of the resulting changepoint estimation then relies on this estimate being sufficiently close to the true value, and so the non-background data fraction must be small (Fisch et al 2018). This is unlikely to hold in our motivating case, when nuisance changes in the background level are possible.

Another option is to define:

$$\begin{aligned} F(n; \theta _0) = \min _{k,\{(s_i, e_i)\}_k, \{\theta _i\}_k} F(n; \{(s_i, e_i)\}_k, \{\theta _i\}_k, \theta _0, k), \end{aligned}$$

which can be minimised using OP or PELT, and then minimise it over $\theta _0$ using gradient descent (Zhao and Yau 2019). The main drawback of this approach is an increase in computation time proportional to the number of steps needed for the outer optimisation (typically $>20$).

## Detection of changepoints with unknown background level

To solve the epidemic model in (4) efficiently when the background is unknown and large proportion of non-background data is possible, we introduce Algorithm 1. Similarly to other epidemic changepoint detectors, the algorithm seeks to minimise the penalised likelihood-based cost (5). This is done using OP in steps 3–12, which involves recalculating the optimal segmentation for each new data point.

The main innovation is that we update the estimate of $\theta _0$ after each point (step 6): if the new data point is determined to come from the background distribution, the estimate is recalculated from the current background points. For full generality, the algorithm is presented with any estimator *g* for the background parameter, although we will focus on the case where *g* is the sample mean or variance. These can be updated using their recursive formulas, which is particularly fast, requiring only ${\mathcal {O}}(1)$ operations at each point.

The iterative updating produces a good estimate in a single pass over the data, and so is more computationally efficient than aPELT (Zhao and Yau 2019), which repeats the entire segmentation with many possible $\theta _0$ values. Because the proposed algorithm simultaneously segments and estimates the background, it is also more accurate and robust than methods that attempt to estimate $\theta _0$ from unsegmented data, such as CAPA (Fisch et al 2018).

The algorithm as stated here includes a second pass over the data (step 13), repeating the segmentation with the final estimate of $\theta _0$. The purpose is to update the changepoint positions that are close to the start of the data and so had been determined using less precise estimates of $\theta _0$. This simplifies the theoretical performance analysis, but an attractive option is to use this algorithm in an online manner, without this step. We evaluate the practical consequences of this omission explicitly in Sect. 5.

The algorithm also includes a search length parameter *l*: this may be set to limit segment duration based on prior knowledge (as will be used later in the paper for signal segments), or kept unlimited, as $l=n$.
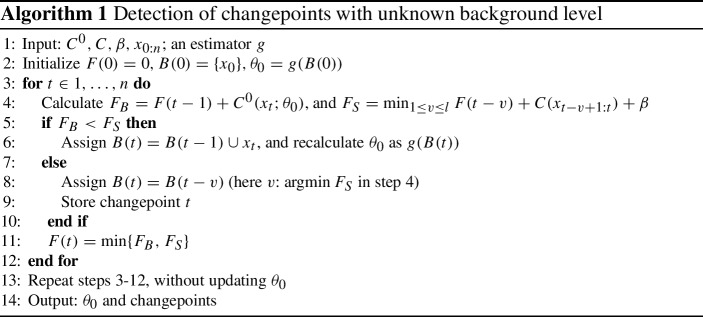


We will demonstrate some theoretical properties of this approach next. In Sect. 5 we investigate its performance with simulations.

### Convergence

The changepoint model can be understood as a function over an interval that is sampled to obtain *n* observed points. We explore the properties of the algorithm as the sampling density increases: in this setting, the number and strength of changes are fixed, but the length of segments grows as *o*(*n*).

#### Theorem 1

Consider the problem of an epidemic change in mean, with data $x_{0:n}$ generated as in (4). Assume the pdf $f_B(x)$ and marginal pdf of segment points $f_S(x)$ are symmetric and strongly unimodal, with unknown background mean $\theta _0$, and that data points within each segment are iid. Denote by $w_t$ the estimate of $\theta _0$ obtained by analysing $x_{0:t}$ by Algorithm 1. The sequence $\{w_t\}$ converges:

1. to the true background value $\theta _0$ almost surely if $\int _{-\infty }^{\infty } x f_S(x) dx = \theta _0$.
2. to a neighbourhood $(\theta _0 - \epsilon , \theta _0 + \epsilon )$ almost surely, where $\epsilon \rightarrow 0$ as the number of background points *n* between successive segments $n\rightarrow \infty$.

We refer the reader to Supplementary Material S1 for the proof. It is based on a result by Bottou (1998), who established conditions in which an online minimisation algorithm almost surely converges to the optimum. We show that in the first case, the updating process in our algorithm satisfies these conditions directly. (This case could arise with various combinations of fixed $\theta _i$, or, for example, if the segment means are modelled as coming from a Gaussian prior centred around $\theta _0$.) In the second case, weak changes may be missed and cause $w_t$ to deviate from $\theta _0$. Then the conditions can be satisfied by defining update cycles comprising points between successive misclassifications. As *n* increases, weaker changes get detected accurately, so the frequency of misclassification drops and convergence improves.

### Consistency for Gaussian data

As the sampling density increases, more accurate estimation of the number and locations of changepoints is expected; this property is formalised as consistency of the detector. Fisch et al (2018) showed that detectors based on minimising penalised cost are consistent for Gaussian data, and their result can be adapted to prove consistency of Algorithm 1. The strengthened SIC penalty $\alpha \log (n)^{1+\delta }$ is used. Additionally, following Fisch et al (2018), we set the following minimum signal strength bound:

6 $$\begin{aligned} \forall i, (e_i-s_i)\Delta _i > \log (n)^{1+\delta }, \end{aligned}$$

where $\Delta _i$ represents the strength of change associated with segment *i*, relative to the background parameters $\mu _0, \sigma _0$:

$$\begin{aligned} \Delta _i = \min (\tilde{\Delta }_i, \tilde{\Delta }^2_i), \text { with } \tilde{\Delta }_i = \log \left( \frac{(\mu _0 - \mu _i)^2 + 2(\sigma ^2 + \sigma ^2_i)}{4\sigma _0 \sigma _i} \right) . \end{aligned}$$

#### Theorem 2

Let the data $x_{0:n}$ be generated from the epidemic changepoint model (4), with $f_B$ and $f_S$ Gaussian, and the changing parameter $\theta$ be either its mean or variance (assume the other parameter is known). Further, assume (6) holds for *k* changepoints. Analyse the data using Algorithm 1 with penalty $\beta = \alpha \log (n)^{1+\delta }$, $\alpha ,\delta >0$. The estimated number and position of changepoints will be consistent, i.e. $\forall \epsilon>0, n>B$:

7 $$\begin{aligned} P\left( \hat{k} = k, \max \{ |\hat{s}_i-s_i |, |\hat{e}_i-e_i |\} < \frac{A}{\Delta _i} \log (n)^{1+\delta }, \forall 1\le i \le k \right) \ge 1 - Cn^{-\epsilon }, \end{aligned}$$

for some *A*, *B*, *C* that do not increase with *n*.

The proof is given in Supplementary Material S2. We use the connection between Algorithm 1 and stochastic gradient descent to establish error bounds on the background parameter estimates. These bounds then allow us to apply a previous consistency result (Fisch et al 2018) to our case.

***Remark*** Theorem 2 still holds if the other parameter is not known, but estimated to a precision of ${\mathcal {O}}\left( \sqrt{\log (n)/n}\right)$. As shown in Fisch et al (2018), if the non-background contamination is limited, this is satisfied by robust estimators such as the interquartile range for scale.

Standard PELT-style pruning can be applied to Algorithm 1, with most likelihood-based costs. We detail the corresponding implementation and show that it does not change the optimisation result in Supplementary Material S3.

## Detecting changepoints with a nuisance process

### Problem setup

In this section, we consider the changepoint detection problem when there is an interfering nuisance process. We assume that this process, like the signal, consists of segments, which we denote by $s^N_j, e^N_j$. Data within these segments is generated from a nuisance-only distribution $f_N$, or from some distribution $f_{NS}$ if a signal occurs at the same time. In total, four states are possible, so the overall model of data is:

8 $$\begin{aligned} f(x_t) = {\left\{ \begin{array}{ll} f_{NS}(x_t; \theta _i, \theta ^N_j) &{} \text { if } \exists i,j: t \in [s_i, e_i] \cap [s^N_j, e^N_j] \\ f_S(x_t; \theta _i) &{} \text { if } \exists i: t \in [s_i, e_i], t \notin \cup _j [s^N_j, e^N_j] \\ f_N(x_t; \theta ^N_j) &{} \text { if } \exists j: t \in [s^N_j, e^N_j], t \notin \cup _i [s_i, e_i] \\ f_B(x_t) &{} \text { otherwise.} \end{array}\right. } \end{aligned}$$

We add two more conditions to ensure identifiability:

1. The nuisance process evolves more slowly than the signal process, so $\min (e^N_j-s^N_j) > \max (e_i-s_i)$.
2. Signal segments are either entirely contained within a nuisance segment, or entirely out of it:
  9 $$\begin{aligned} \forall i,j, \text { either } [s_i, e_i] \subset (s^N_j, e^N_j), \text { or } [s_i, e_i] \cap [s^N_j, e^N_j] = \emptyset . \end{aligned}$$

***Remark*** In practice, there should be a sufficient gap between these lengths to allow for error in *s*, *e* estimates, typically of order $o(\log n)$. This is satisfied in our setting where the changepoint positions are fixed. Violating the second condition produces several shorter segments which cannot be unambiguously resolved, but in practice will result in one or more detections near the true signal segment and thus is not a major obstacle.

To define the penalised cost of such a model, let $X_S = \bigcup _i x_{s_i:e_i}$, $X_N = \bigcup _j x_{s^N_j:e^N_j}$, set penalties $p(k)=\beta k$, $p'(m) = \beta 'm$ for the numbers of signal and nuisance segments, respectively, and cost functions $C^{NS},~C^S,~C^N,~C^0$ corresponding to the log-likelihoods of each of the distributions in (8). Then the full cost is:

10 $$\begin{aligned}&F(n; \{(s_i, e_i)\}_k, k, \{(s^N_j, e^N_j)\}_m, m, \varvec{\theta }) = C^0(x_{0:n} \setminus (X_S \cup X_N) ) + \sum _{i=0}^k C^S(x_{s_i:e_i} \setminus X_N; \theta _i) \nonumber \\&\quad + \sum _{j=0}^m \left( C^N(x_{s^N_j:e^N_j} \setminus X_S; \theta ^N_j) + \sum _{i=0}^k C^{NS}(x_{s^N_j:e^N_j} \cap x_{s_i:e_i}; \theta _i, \theta ^N_j) \right) + \beta k + \beta ' m. \end{aligned}$$

### Proposed method

To minimise the cost in (10), first notice that it can also be expressed, using $k_j$ as the number of signal segments that overlap a nuisance segment *j* and $k_0 = k - \sum k_j$, as:

$$\begin{aligned} F(n; \{(s_i, e_i)\}_k, k, \{(s^N_j, e^N_j)\}_m, m, \varvec{\theta }) = C^0(x_{0:n} \setminus (X_S \cup X_N) ) \\ + \sum _{i=0}^{k_0} C^S(x_{s_i:e_i} \setminus X_N; \theta _i) + \beta k_0 + \sum _{j=0}^m (C'(x_{s^N_j:e^N_j}) + \beta '). \end{aligned}$$

with $C'(\cdot ) = F(\cdot ; \{(s_i, e_i)\}_{k_j}, \{\theta \}_{k_j}, k_j)$, the standard epidemic cost in (5). That is, over each proposed nuisance segment $s^N_j:e^N_j$, an epidemic changepoint problem with unknown background parameter must be solved. Condition (9) ensures that $C', C^S, C^0$ are independent (do not share any points or parameters), so *F* is block-additive and can be minimised by OP.

A method to minimise this cost is outlined in Algorithm 2. In it, an outer loop proceeds over the data to identify segments by the usual OP approach. Length bound *l* determines what type of cost is applied: signal segment cost $C^S$, or nuisance (with potential signals) cost $C'$. If $C'$ is needed, it is minimised in an inner loop using Algorithm 1. This is a key difference from related methods: evaluating more complex cost $C'$ is only possible because of the efficiency of Algorithm 1. Thus, the parameter *l* allows incorporating prior knowledge about signal duration to distinguish segment types, and should ideally be set so that $\min (e^N_j-s^N_j)> l > \max (e_i-s_i)$.

By Theorem 2, this process will estimate the number and positions of true segments consistently given accurate assignment of $s^N_j, e^N_j$. However, the latter event is subject to a complex set of assumptions on relative signal strength, position, and duration of the segments. Therefore, we do not attempt to describe these in full here, but instead investigate the performance of the method by extensive simulations in Sect. 5.3.
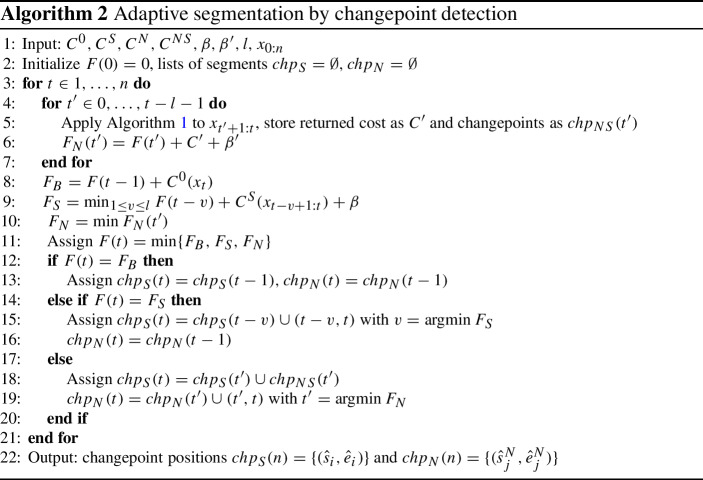


Algorithm 2 is stated assuming that a known or estimated value of the parameter $\theta _0$, corresponding to the background level without the nuisance variations, is available. In practice, it may be known when there is a technical noise floor or a meaningful baseline that can be expected after removing the nuisance changes. Alternatively, $\theta _0$ may be substituted by a robust estimate, or the method can be modified to estimate it simultaneously with segmentation, using a principle similar to Algorithm 1. No relation between the distributions $f_B, f_S, f_N, f_{NS}$ is assumed, although in subsequent sections we will mostly focus on the challenging case when both signal and nuisance changes affect the same parameter.

### Pruning

In the proposed method, the estimation of the mean of segment *j* is sensitive to the segment length, therefore the cost $C'$ is not necessarily block-additive (3), and so it cannot be guaranteed that PELT-like pruning will be exact. However, we can establish a local pruning scheme that retains the exact optimum with probability $\rightarrow 1$ as $n \rightarrow \infty$.

#### Proposition 1

Assume data $x_{0:n}$ is generated from a Gaussian epidemic changepoint model, and that the distance between changepoints is bounded by some function *A*(*n*):

$$\begin{aligned} \forall i,j,j': \min \{ |s^N_j - e^N_{j'} |, |s_i - s^N_j |, |e_i - e^N_j |\} > A(n). \end{aligned}$$

At time *t*, the solution space is pruned by removing:

11 $$\begin{aligned} \varvec{k}_{pr, t} = \{k : F(k-1) + C'(x_{k:t}) \ge \min _m F(m-1) + C'(x_{m:t}) + \alpha \log (n)^{1+\delta } \}. \end{aligned}$$

Here $m\in (t-A(n); t], k \in (t-A(n); t], k \ne m$. Then $\forall \epsilon >0$, there exist constants $B, n_0$, such that when $n>n_0$, the true nuisance segment positions are retained with high probability:

$$\begin{aligned} P\left( \forall j: s^N_j \notin \bigcup _t \varvec{k}_{pr, t} , e^N_j \notin \bigcup _t \varvec{k}_{pr, t}\right) \ge 1-Bn^{-\epsilon }. \end{aligned}$$

The proof is given in Supplementary Material S4. The assumed distance bound serves to simplify the detection problem: within each window $(t-A(n), t]$, at most 1 true changepoint may be present, and the initial part of Algorithm 2 is identical to a standard epidemic changepoint detector. It can be shown that other candidate segmentations in the pruning window are unlikely to have significantly lower cost than the one associated with $s^N_j, e^N_j$, and therefore $s^N_j, e^N_j$ are likely to be retained in pruning.

This scheme only prunes within windows of user-set size *A*(*n*) and so is less efficient. By choosing large *A*(*n*), the efficiency can be increased, but that may violate the data generation model and cause some true changepoints to be lost. However, assuming that the overall estimation of nuisance changepoints is consistent, Proposition 1 extends to standard pruning over the full dataset. We show that this holds empirically in Sect. 5.3.

## Simulations

In this section, we present the simulations used to evaluate the performance of the methods. The corresponding R code is available at https://github.com/jjuod/changepoint-detection.

### Algorithm 1 estimates the background level consistently

Firstly, we tested that Algorithm 1 estimates the background parameter accurately (Theorem 1). Datasets were generated under three different scenarios with changes in mean:

**One segment** Gaussian with one signal segment: *n* data points were drawn as $x_t \sim {\mathcal {N}}(\theta _t, 1)$, with $\theta _t = 3$ at times $t \in (0.3n; 0.5n]$, and 0 otherwise (background level).

**Multiple** Gaussian with multiple signal segments: *n* data points were drawn as $x_t \sim {\mathcal {N}}(\theta _t, 1)$, with:

$$\begin{aligned} \theta _t = {\left\{ \begin{array}{ll} -1 \text { when } t \in (0.2n; 0.3n] \cup (0.7n; 0.8n] \\ 1 \text { when } t \in (0.5n; 0.6n] \\ 0 \text { otherwise.} \end{array}\right. } \end{aligned}$$

**Heavy tail** Heavy tailed data with one signal segment: *n* data points were drawn from the generalized *t* distribution as $x_t \sim T(3) + \theta _t$, with $\theta _t = 2$ at times $t \in (0.2n; 0.6n]$ and 0 otherwise.

We generated time series for each of the three scenarios with values of *n* between 30 and 750, with 500 replications for each *n*. Note that the segment positions remain the same for all sample sizes, so the increase in *n* could be interpreted as denser sampling of the underlying function.

Each time series was analysed using Algorithm 1 to estimate $\theta _0$. The maximum segment length was set to $l=0.5n$ and the penalty to $\beta =3 \log (n)^{1.1}$. The cost was computed using the Gaussian log-likelihood function with the true variance value. This means that the cost function was mis-specified in Scenario 3, and so provides a test of the robustness of the algorithm (although the variance was set to 3, as expected for *T*(3)).

For comparison, we analysed the data using the R package *anomaly* (Fisch et al 2018), which estimates $\theta _0$ as the median of the entire time series $x_{1:n}$. The method allows separating segments of length 1, but we treated them as standard segments. We also implemented the profile *aPELT* algorithm (Zhao and Yau 2019) by adding an optimisation loop over the possible $\theta _0$ values to a PELT-style detector. The original paper also allows for series starting in a segment, which we did not implement as it is not the case in our simulations. Both comparison methods used the same maximum length, penalty and background variance values as Algorithm 1. As the oracle efficiency limit, based on the CLT, we show the quantiles of ${\mathcal {N}}(0, \sigma ^2/\sqrt{n_B})$, with $n_B$ the total number of background points.

**Fig. 1:**
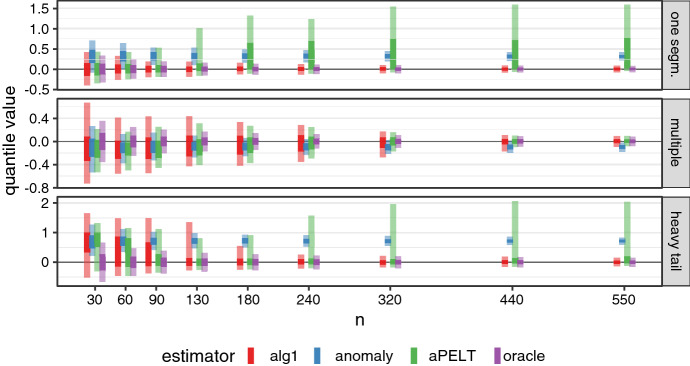
Consistency of the background level estimation. Time series simulated in three different scenarios were analysed by Algorithm 1 (shown in red). Lines are the inter-quartile range (solid) and 5–95% range (faint) of the background parameter estimates observed in 500 replications. For comparison, we show the ranges of background estimates obtained from two other segmentation algorithms and an oracle estimator (mean of true background points)

As seen in Fig. 1, Algorithm 1 produced consistent and efficient estimates of the background level. The *anomaly* estimator, based on non-segmented data, was often biased, although may be preferrable at low *n*, where other estimates showed large variance. The optimization of *aPELT* sometimes settled in local minima in which the background was estimated entirely from non-background points, causing major errors even at large sample sizes. At $n>400$, our algorithm provides the lowest total error and near-oracle performance in all tested scenarios, even with the mis-specified cost function in the *heavy tail* scenario.

### Segment positions are accurately estimated by Algorithm 1

The same setup was used to evaluate the consistency of estimated segment number and positions. From the simulations described above, we extracted the mean number of segments reported by each method, and also calculated the true positive rate (TPR) as the fraction of simulations in which the method reported at least 1 changepoint within 0.05*n* points of each true changepoint.

In all three scenarios, the TPR for the proposed Algorithm 1 approaches 1 (Table 1). When the signal is strong (*one segment* scenario), the segmentation was accurate even at $n=30$. In scenario *heavy tail*, the algorithm correctly detected changes at the true segment start and end, but tended to fit the segment as multiple ones, due to the heavy tails of the *t* distribution. The comparison methods performed similarly to ours in the *multiple* scenario, but showed more false alarms or lower TPR in the other two scenarios. This is consistent with their estimation results seen in Fig. 1, and highlights the importance of accurately determining the background for good segmentation.

**Table 1:** Consistency of the estimated changepoints

Scenario	*n*	Mean # seg.			TPR		
		Alg. 1	Anomaly	aPELT	Alg. 1	Anomaly	aPELT
One segm.	30	1.12	1.15	1.10	0.916	0.942	0.934
	90	1.06	1.25	1.16	0.998	0.998	1.000
	180	1.04	1.42	1.66	0.996	1.000	0.986
	440	1.03	2.13	2.06	0.994	1.000	0.986
	750	1.01	2.68	2.27	0.998	1.000	0.990
Multiple	30	0.53	0.37	0.29	0.000	0.002	0.002
	90	1.12	1.06	1.03	0.010	0.022	0.008
	180	1.83	1.99	1.86	0.128	0.168	0.124
	440	2.89	2.97	2.96	0.814	0.866	0.868
	750	3.02	3.02	3.02	0.982	0.972	0.984
Heavy	30	0.66	0.56	0.47	0.124	0.080	0.082
	90	1.41	1.45	1.38	0.594	0.510	0.646
	180	1.86	2.17	1.88	0.860	0.766	0.846
	440	2.83	3.70	3.07	0.984	0.930	0.858
	750	3.86	5.10	4.09	1.000	0.986	0.840

Time series simulated in three different scenarios were analysed using Algorithm 1 or two other epidemic detectors, in 500 replications for each *n*. The mean number of reported segments and the TPR (fraction of replications when a changepoint was detected within 0.05*n* of each true changepoint) are shown. The number of true segments was 1, 3, and 1 for the *one segm.*, *multiple* and *heavy* scenarios, respectively

We also retrieved the changepoint positions that were estimated in step 12 of Algorithm 1. This corresponds to its online usage, in which segmentation is not repeated after the first pass over the data. This had very little impact on the result accuracy (Table ST1 in Supplementary Material S6), suggesting that this simplification can be safely used.

Finally, in each iteration we also calculated the cost *F*(*n*) (i.e., log-likelihood of the fitted Gaussian changepoint model, with penalty $\beta$) to evaluate if the algorithm achieves the stated optimization objective. The minimum reached by Algorithm 1 was as good as, or even smaller than, the cost based on true segmentation (Table ST2 in Supplementary Material S6). Very little differences between the full and online versions of the algorithm were seen.

### Algorithm 2 recovers true segments under interference

We generated time series under three different scenarios. In each, points $x_{1:n}$ were drawn from a Gaussian distribution with changes in mean. Series were generated for *n* between 30 and 220, in 1000 replications at each *n*.

**Scenario 1** A signal segment overlapping a nuisance segment: $x_t \sim {\mathcal {N}}(\theta ^S_t + \theta ^N_t, 1)$, with $\theta ^S_t = 2$ when $t \in (0.3n; 0.5n]$, 0 otherwise, and $\theta ^N_t = 2$ when $t \in (0.2n; 0.7n]$, 0 otherwise.

**Scenario 2** A nuisance segment and two non-overlapping signal segments: $x_t \sim {\mathcal {N}}(\theta ^S_t + \theta ^N_t, 1)$, with:

$$\begin{aligned} \theta ^N_t = 1.5 \text { when } t \in (0.2n; 0.4n],~0 \text { otherwise.} \\ \theta ^S_t = {\left\{ \begin{array}{ll} 3 \text { when } t \in (0.5n; 0.6n] \\ -3 \text { when } t \in (0.7n; 0.8n] \\ 0 \text { otherwise.} \end{array}\right. } \end{aligned}$$

**Scenario 3** Many weak signal segments: $x_t \sim {\mathcal {N}}(\theta ^S_t, 1)$, $\theta ^S_t = \theta _j$ when $t/n \in (0.1j; 0.1j+0.05]$ for $j=1,\dots ,9$; 0 otherwise. Segment means are random, $\theta _j \sim \mathrm {Unif}(-4,4)$.

Each series was analysed by five methods. Besides Algorithm 2 (*proposed*), we used the epidemic detectors *anomaly* and *aPELT* as in the simulations above. All penalties for these methods were set to $3\log (n)^{1.1}$. The *sparse* method is an epidemic detector designed for sparse segments with a known background level (Jeng et al 2010). It performs a greedy search over all possible intervals up to length *l*. We used a penalty of $\sqrt{2\log (nl)}$, recommended by the original publication. All of these methods allow a maximum segment length, which we set to $l=0.33n$ in scenario 1, 0.15*n* in scenario 2, 0.2*n* in scenario 3. Global background parameters $\mu _0=0, \sigma =1$ were also provided to all methods (although *aPELT* will re-estimate $\mu _0$ on its own).

As an example of a different approach, we included the narrowest-over-threshold detector implemented in R package *not*, with default parameters. This is a non-epidemic changepoint detector that was shown to outperform most comparable methods (Baranowski et al 2019). Since it does not include the background-signal distinction, we define signal segments as regions between two successive changepoints where the mean exceeds $\mu _0 \pm \sigma _0$.

For evaluation, we declared a true positive if a changepoint of the correct type (i.e., start or end of a signal segment) was reported within 0.05*n* points of a true changepoint. Using these, the true positive rate and mean localization error were calculated as defined in the Supplementary Material. We also calculated positive predicted value (PPV) as the fraction of true positives among all the reported segments: higher values of this metric correspond to lower false discovery rate, or e.g., lower review effort if the reported segments would be subject to further manual inspection. Furthermore, in scenario 1, we extracted the estimate of $\theta$ corresponding to the detected segment (or the one closest to the true position if multiple were detected). The average of these over the replications was reported as $\hat{\theta }^S$.

We also compared Algorithm 2 when applied without pruning, or with global pruning as in (11), with $m\in (0; t-l)$ at each *t*. In scenarios 1 and 2, pruning changed the result in only 4 out of 10,000 runs; some more differences (in $\le \,2$% of the runs) were observed in scenario 3. As the results were mostly identical, we only present those obtained with pruning from here on.

**Fig. 2:**
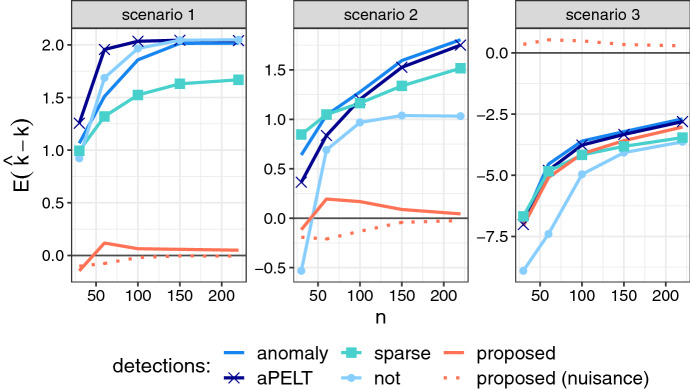
Relative bias in the number of changepoints estimated by the proposed Algorithm 2 (pruned), and four alternative detectors. Data simulated in 1000 replications. For the proposed algorithm, bias is calculated separately in signal (solid line) and nuisance (dashed) segments

We observed that the proposed method (with pruning) successfully detected true signal segments, with much fewer false positives than the other methods (Fig. 2). The number of nuisance detections was accurate in scenario 1, and slightly underestimated in favour of more signal segments in scenario 2, most likely because the simulated nuisance length was close to the cutoff *l*. As expected, the reference methods that do not include nuisance segments in the model identify them as multiple changepoints; as a result, the number of segments was over-estimated up to 3-fold (Fig. 2).

We verified that the proposed method reported the number and position of the true segments as accurately as the other methods. There were no major differences in absolute localization error or the fraction of true changepoints captured between the proposed and the top methods (Supplementary Tables S3, S4). This was also specifically tested in scenario 3: it had no nuisance segments, but the ability to estimate them did not harm the *proposed* method, and it performed on par with the best detectors (Fig. 2). The short and weak segments here were difficult to detect in general, and *not* performed consistently worse, showing the benefits of utilising the background structure, especially at smaller sampling densities.

Thus, the main practical benefit of the proposed method is the much smaller number of false alarms per true detection. This is summarized by the PPV metric in Table 2, which shows that our method consistently outperforms the others throughout scenarios 1 and 2, and is on par in scenario 3. In addition, the other models are also unable to capture the signal-specific change in mean $\theta ^S$: *anomaly* estimated $\hat{\theta }^S=4.00$, and *not* estimated $\hat{\theta }^S=3.98$ for the segment in scenario 1 at $n=220$. These values correspond to the sum of the signal and nuisance effects. While the estimation is accurate and could be used to recover the signal-specific change by post-hoc analysis, our proposed method estimated it directly, as $\hat{\theta }^S=2.01$ in scenario 1 at $n=220$.

**Table 2:** The positive predictive value (PPV) of changepoint estimation by the proposed Algorithm 2 and alternative detectors

Scenario	*n*	Proposed	anomaly	aPELT	sparse	not
1	30	**0.618**	0.329	0.337	0.300	0.315
	60	**0.719**	0.297	0.303	0.203	0.304
	150	**0.940**	0.330	0.329	0.180	0.328
	220	**0.950**	0.331	0.329	0.161	0.330
2	30	**0.868**	0.731	0.805	0.691	0.639
	60	**0.878**	0.655	0.706	0.653	0.708
	150	**0.955**	0.560	0.572	0.598	0.666
	220	**0.975**	0.526	0.535	0.569	0.663
	30	**0.994**	0.992	0.993	0.989	0.819
	60	0.892	**0.985**	0.979	0.983	0.787
	150	**1.000**	0.999	0.999	0.999	0.996
	220	0.968	**0.998**	0.997	**0.998**	0.987

Data simulated in 1000 replications. The PPV is the number of reported changepoints that are correct (within 0.05*n* of a true signal changepoint) divided by the total number of reported detections. The best results in each row are highlighted

As the length bound parameter *l* is key to separating the segment types in our method, but likely will not be precisely known in practice, we repeated the scenario 1 simulations with different *l* values. The *proposed* algorithm was not particularly impacted in the range of values tested, and consistently produced around 1 signal segment and a TPR of 0.70–0.71 (Supplementary Table S5). The other epidemic detectors were affected more, in particular the *sparse* method, for which the TPR dropped from 0.54 to 0.07 when *l* was increased from 0.25*n* to 0.33*n*. This example highlights the potential dangers of using greedy algorithms when overlapping segments are present. Note however that the tested *l* values are overestimates of the maximum signal length: otherwise any signal segments exceeding the specified *l* will be identified as nuisances by the *proposed* method, and the detection power reduced correspondingly.

## Real-world data

In this section, we present the results of real data analysis using the proposed method. Data sources and additional experiment details are presented in Supplementary Material S5. The corresponding R code is available at https://github.com/jjuod/changepoint-detection.

### ChIP-seq

As an example application of the algorithms proposed in this paper, we demonstrate peak detection in chromatin immunoprecipitation sequencing (ChIP-seq) data. The goal of ChIP-seq is to identify DNA locations where a particular protein of interest binds, by precipitating and sequencing bound DNA. This produces a density of binding events along the genome that then needs to be processed to identify discrete peaks. Typically, some knowledge of the expected peak length is available to the researcher. Furthermore, the background level may contain local shifts of various sizes, caused by sequencing bias or true structural variation in the genome (Zhang et al 2008). The method proposed in this paper is designed for such cases, and can potentially provide more accurate and more robust detection.

**Fig. 3:**
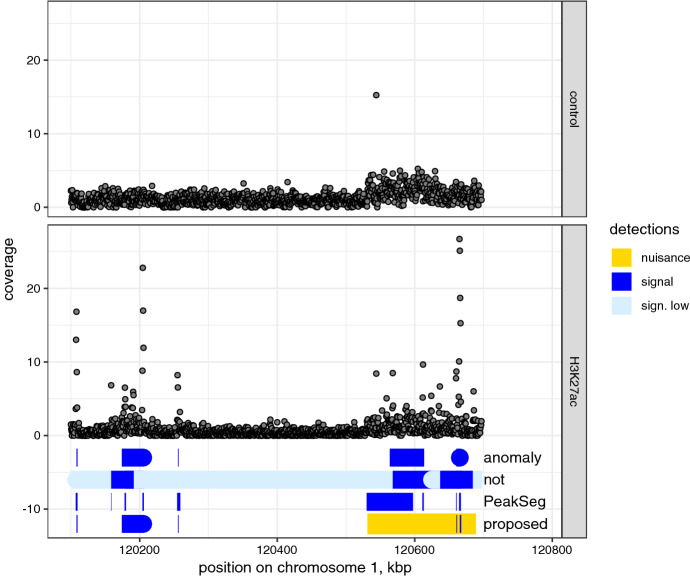
ChIP-seq read counts and analysis results. Counts provided as mean coverage in 500 bp windows for a non-specific control sample (top) and H3K27ac histone modification (bottom), chromosome 1. Segments detected in the H3K27ac data by the method proposed here (Algorithm 2) and three other detectors are shown under the counts. Note that the proposed method can produce longer nuisance changes overlapped by signal segments. *not* does not specifically identify background segments; we show the ones with relatively low mean in light colour

We used two binding density series obtained in ChIP-seq experiments (see Supplementary Material S5 for details). Broad Institute H3K27ac data is not annotated, but includes a control track which reveals the presence of some nuisance variation (Fig. 3, top). UCI/McGill dataset was previously used for evaluating peak detectors (Hocking et al 2017, 2018). It includes annotations based on visual inspection. These are weak in the sense that they indicate peak presence or absence in a region, not their exact positions, to acknowledge the labelling subjectivity. The series were analysed by the proposed Algorithm 2, *anomaly*, *not*, and *PeakSegDisk* (Hocking et al 2018), a changepoint detector developed specifically for ChIP-seq data. The penalties of all four detectors were calibrated to similar sensitivity, using the Broad Institute control track (see Supplementary Material S5). Note that the proposed algorithm was used with a cost based on Gaussian likelihood, as presented so far, even though the data are positive counts.

In the H3K27ac data, all methods detected the two most prominent peaks, but produced different segmentations for smaller peaks and more diffuse change areas (Fig. 3, bottom). All three reference detectors marked a broad segment in the area around 120,600,000 bp. Based on comparison with the control data, this change is spurious, and it exceeds the 50 kbp bound set for target segments. While this bound was provided to the *anomaly* detector, it does not include an alternative way to model these changes, and therefore still reports one or more shorter segments. In contrast, our method accurately modelled the area as a nuisance segment with two overlapping sharp peaks, even with data clearly deviating from the assumed Gaussian model.

Using *not*, the data was partitioned into 16 segments. By defining segments with low mean ($\theta < \hat{\mu }_0 + \hat{\sigma }_0$) as background, we could reduce this to 8 signal segments; however, then the short peaks around 120,200,000 bp are missed, which fit the definition of signal ($<50$ kbp) and were retained by the proposed method. This data illustrates that choosing the post-processing required for most approaches is not trivial, and can have a large impact on the results. In contrast, the parameters required for our method have a natural interpretation and may be known a priori or easily estimated, and the outputs are provided in a directly useful form.

**Fig. 4:**
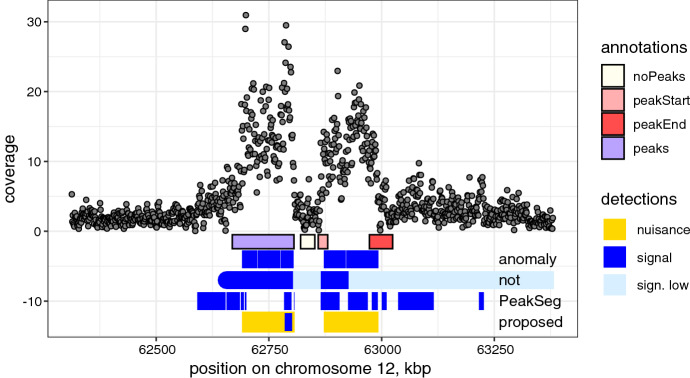
UCI/McGill ChIP-seq data: read coverage in 1100 bp windows, black points, and manual annotations of peaks (boxes). Detection results using Algorithm 2 proposed in this paper, as well as three state-of-the-art methods are shown at the bottom

In the UCI data, segment detections also generally matched the visually determined labels (Fig. 4). However, our method produced the most parsimonious models to explain the changes, reporting two nuisance segments and a single sharp peak around 62,750,000 bp. The nuisance segments correspond to broad regions of mean shift, which were also detected by *anomaly* and *not*, but using 6 and 7 segments, respectively. Notably, *PeakSeg* differed considerably: as this method does not incorporate a single background level, but requires segments to alternate between background and signal, the area around 62,750,000 bp was defined as background, despite having a mean of $4.5~\hat{\mu }_0$. In total, 12 segments were reported by this method. This shows that the ability to separate nuisance and signal segments helps produce more parsimonious models, and in this way minimises the downstream efforts such as experimental replication of the peaks.

The visual annotations provided for this region are shown in the first row in Fig. 4. Note that they do not distinguish between narrow and broad peaks (single annotations in this sample range up to 690 kb in size). Furthermore, comparison with such labels does not account for finer segmentation, coverage in the peak area, or the number of false alarms outside it. For these reasons we are unable to use the labels in a quantitative way.

For a quantitative comparison of the detectors, we use SIC. The proposed method is favoured in the Broad dataset, producing SIC of 3644, while *PeakSeg*, *not* and *anomaly* had an SIC of 4474, 22251, and 3781, respectively. The smallest SIC values in UCI data were also produced by *anomaly* (5012) and our method (5045), while *not* resulted in an SIC of 14572 and *PeakSeg* 8339. Thus, in addition to the practical benefits, the nuisance-signal structure can provide a better fit to these series than models that allow only one type of segments.

### European mortality data

The recent pandemic of coronavirus disease COVID-19 prompted a renewed interest in early outbreak detection and quantification. In particular, analysis of mortality data provided an important resource for guiding the public health responses to it (e.g., Baud et al 2020; Yuan et al 2020).

We analysed weekly mortality in 60–64 year age group in Spain over a four year period, using the four methods introduced earlier. Besides the impact of the pandemic, this data contains longer nuisance increases corresponding to winter seasons. As before, Gaussian likelihood was assumed for the proposed Algorithm 2.

**Fig. 5:**
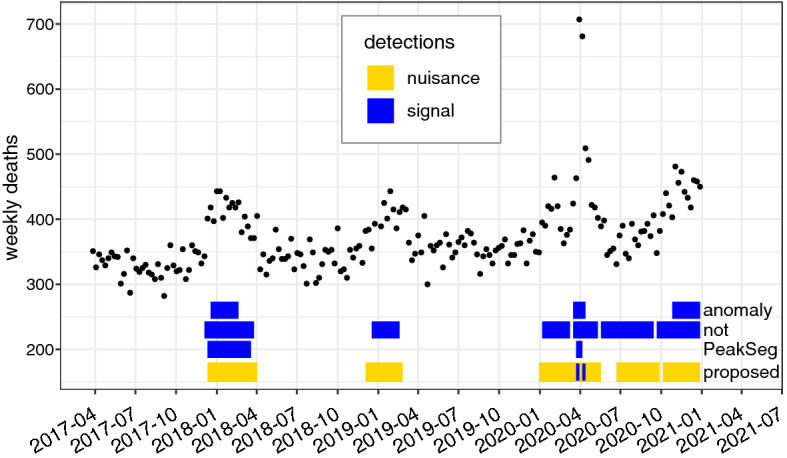
Weekly deaths in Spain, in the 60–64 years age group, over 2017–2020 (black points). Detection results using the method proposed in this paper and three alternative methods shown as lines below

The detected segments are shown in Fig. 5. Three of the methods, *anomaly*, *PeakSeg*, and Algorithm 2, detected the sharp spikes around the pandemic period. However, *anomaly* and *PeakSeg* also marked one winter period as a signal segment, while ignoring the others. Six segments were created by *not*, including a broad peak extending past the bounds of the pandemic spike. In contrast, the proposed method marked two sharp pandemic spikes, while also labelling all winter periods as nuisance segments. The resulting detection is again parsimonious and flexible: if only short peaks are of interest, our method reports those with lower false alarm rate than the other methods, but broader segments are also marked accurately and can be retrieved if relevant. It should be noted that the data also contains a secular increasing trend, and our method reports an additional nuisance segment in 2020. Such trends or periodic patterns, if expected in the data, could be removed e.g. by applying an ARMA model, and then changepoint detectors could be more readily applied to the residuals.

As in the ChIP-seq data, comparing the results by SIC identifies our method as optimal for this dataset (SIC of 1898 for Algorithm 2 vs. 2032, 2189, and 2388 for the other methods). Note that the SIC penalizes both signal and nuisance segments, so in this case our model still appears optimal despite having more parameters.

## Discussion

In this paper, we have presented a pair of algorithms for improving detection of epidemic changepoints. Similarly to stochastic gradient descent, the iterative updating of the background estimate in Algorithm 1 leads to fast convergence while allowing large fraction of non-background points. This is utilised in Algorithm 2 to analyse nuisance-signal overlaps. We have shown in the simulations that the algorithms outperform state-of-the-art methods, producing better background estimates and fewer false positives. While the simulations and theoretical analysis focus on the specific model of Gaussian data and step-like changes, practical results show that the detection is robust and can be usefully applied to data that strongly deviates from these assumptions.

With the PELT-style pruning, the computational complexity of Algorithm 2 is ${\mathcal {O}}(n)$ in the best case, which is similar to state-of-the-art methods (Killick et al , 2012; Hocking et al , 2018). However, this is stated in the number of required evaluations of the segment cost function *C*. It is usually implicitly assumed that *C* is recursive, so that adding each new data point requires only ${\mathcal {O}}(1)$ operations. In our case, evaluating *C* is itself a segmentation task, and this constraint would not be achievable with segmenters that require many passes over the data, such as aPELT (Zhao and Yau 2019) or even more complex ones (Ma et al 2020). The online form of Algorithm 1 was thus essential to create the overlap detector: based on PELT, it can be updated recursively in ${\mathcal {O}}(1)$ time, and allows running the full Algorithm 2 in linear time.

While many simpler methods for classic changepoint detection are available, models incorporating the epidemic structure are more accurate in weak-signal settings, as seen in our simulations. Furthermore, the epidemic methods directly output periods of interest without manual post-processing. This is necessary, for example, in speech detection (Mesaros et al 2017) or observatory data analysis (McNabb et al 2004), where the identified segments are further processed automatically. However the major practical benefit of this framework is the ability to define and separate non-target segments, using only univariate data, as used in this paper. With multidimensional data, if the nuisances affect only a known subset of the variables, it may be possible to use these to estimate and remove the nuisances first, similar to Gao et al (2018). More generally, a common strategy for multidimensional changepoint detection is to first map such data to lower-dimensional series, e.g. by projection (Grundy et al 2020) or summing univariate contrast statistics over the dimensions (Zhang et al 2010). This produces a univariate series which can then be analysed by standard algorithms, including ours, in a variety of situations.

We anticipate that the nuisance-signal separation will aid downstream processing, reducing the false alarm rate or the manual load if the detections are reviewed. Despite that, it is difficult to evaluate this benefit at present: while there are recent datasets prepared specifically for testing changepoint detection (Hocking et al 2017; van den Burg and Williams 2020), they are based on labelling all visually apparent changes. In future work, further application-specific comparisons could measure the impact of neutralising the nuisance process.

## Supplementary Information

Below is the link to the electronic supplementary material.

Supplementary file 1 (pdf 271 KB)

## Data Availability

The data is publicly available with download instructions provided in the Supplementary Material.

## Code Availability

The R code for simulations and data analysis is available at https://github.com/jjuod/changepoint-detection.
