# Supplementary material for: Epidemic changepoint detection in the presence of nuisance changes
Source: Stat Pap (Berl). 2022 Apr 4;64(1):17–39. doi: 10.1007/s00362-022-01307-x (PMC8977442; doi:10.1007/s00362-022-01307-x)
Supplement: Supplementary file 1 — (pdf 271 KB) [file 362_2022_1307_MOESM1_ESM.pdf]

Epidemic changepoint detection in the presence of nuisance changes

Supplementary Material

Julius Juodakis and Stephen Marsland

S1 Proof of Theorem 1 (Convergence of Algorithm 1)

Bottou (1998) analyses the case of an online algorithm iteratively minimising some function $f(x, w)$ (where x represents the complete data and w the parameters). Data points $\{x_t\}$ arrive sequentially, and at each iteration an estimate w_t of the location of the minimum w^* is obtained using some update function $H(x, w)$ and learning rate γ_t as:

$$w_{t+1} = w_t - \gamma_t H(x_{t+1}, w_t). \tag{S1.1}$$

This updating mechanism gives rise to stochastic gradient descent if $\mathbb{E}H(x_{t+1}, w_t) = \nabla_w f(x, w)$, but for the following argument this is not required.

To make the link with Algorithm 1 explicit, the update equation applied by this algorithm can be written as:

$$w_{t+1} = w_t + \gamma_t(x_{t+1} - w_t).$$

Then $w^* = \theta_0$ (i.e., the background mean that is to be estimated), and we ask whether the sequence of updates converges $w_t \to w^*$. It was shown by Bottou (1998) that this occurs almost surely if the following three conditions are met:

1. "convexity" – a single optimum w^* exists and the expected value of the updates always points towards it:

$$\forall \epsilon > 0, \inf_{(w-w^*)^2>\epsilon} (w - w^*)\mathbb{E}H(x, w) > 0; \tag{S1.2}$$

2. learning rate convergence:

$$\sum_{i=1}^{\infty} \gamma_t = \infty \ , \ \sum_{i=1}^{\infty} \gamma_t^2 < \infty; \tag{S1.3}$$

3. bounded variance of the updates:

$$\mathbb{E}H(x,w)^2 \le A + B(w-w^*)^2 \ , \ A, B \ge 0. \tag{S1.4}$$

Thus, proof of convergence of our algorithm reduces to showing that these requirements are satisfied. We start with the assumption that the global mean of segment points is also θ_0, and then relax this requirement.

The following lemma will be needed:

Lemma 1. *Let f be a unimodal pdf, symmetric around a point μ (so that $f(x_1) < f(x_2)$ when $x_1 < x_2 \le \mu$ and $f(x_1) > f(x_2)$ when $\mu \le x_1 < x_2$), such as a Gaussian. Consider a truncated random variable X with pdf:*

$$g(x) = \begin{cases} 0 & \text{if } x < m-a \\ \frac{f(x)}{P(m-a \le x \le m+a)} & \text{if } m-a \le x \le m+a \\ 0 & \text{if } x > m+a \end{cases}$$

for some $a > 0, m$. Then for any $\epsilon > 0$, $\inf_{|m-\mu|>\epsilon}(\mathbb{E}X - m)(\mu - m) > 0$.

Proof.

$$\begin{aligned} \mathbb{E}X - m &= \int_{m-a}^{m+a} (x-m)f(x)dx \\ &= \int_{-a}^{0} yf(y+m)dy + \int_0^a yf(y+m)dy \\ &= \int_0^a y(f(m+y) - f(m-y))dy. \end{aligned} \tag{S1.5}$$

When $m+a < \mu$, f is increasing throughout the integration range, and $\mathbb{E}X - m > 0$; the opposite is true for $m - a > \mu$. If $m - a < m < \mu < m + a$, split the integral in (S1.5) as:

$$\mathbb{E}X - m = \int_0^{\mu-m} y(f(m+y) - f(m-y))dy + \int_{\mu-m}^{a} y(f(m+y) - f(m-y))dy.$$

The first integral covers the range where f is increasing, and thus is positive. Since $\mu - m > 0$, $|m+y-\mu| < |m-y-\mu|$ for $y > 0$, and $f(m+y) > f(m-y)$ by symmetry of f around μ and monotonicity, so the second interval is positive as well. Similarly, $\mathbb{E}X - m < 0$ for $m - a < \mu < m < m + a$. □

S1.1 When the global mean of segments matches the background mean

Consider the case that the background points are independent draws from $\mathcal{N}(w^*, \sigma^2)$, and the points within each segment are $\mathcal{N}(\theta_i, \sigma^2)$, with σ^2 known, and θ_i such that the marginal pdf f_S is symmetric around w^*. Let w_t be the value of the background mean estimated by Algorithm 1 after processing t data points. In this case $w_t \xrightarrow{a.s.} w^*$.

Proof. Denote the true class of the next data point x_{t+1} by δ_{t+1} (1 for background points, 0 for signal). Algorithm 1 estimates this as:

$$\hat{\delta}_{t+1} = \begin{cases} 1 & \text{if } F(t) + C^0(x_{t+1}; w_t) < \min_{1 \le k \le l} F(t+1-k) + C(x_{t+2-k:t+1}) + \beta \\ 0 & \text{otherwise.} \end{cases}$$

Initially, assume for simplicity that the true maximum segment length is 1 (and so only $k = 1$ is tested). When $\hat{\delta}_{t+1} = 1$, the background estimate is updated as:

$$w_{t+1} = w_t + \frac{1}{\sum_{i=1}^{t} \hat{\delta}_i + 1}(x_{t+1} - w_t)$$

(otherwise $w_{t+1} = w_t$). So $\gamma_t = 1/(\sum_{i=1}^{t} \hat{\delta}_i + 1)$, and hence the learning rate convergence conditions (S1.3) are satisfied.

Substituting in the costs based on a one-dimensional Gaussian pdf ϕ, $\hat{\delta}_{t+1} = 1$ if:

$$\begin{aligned} & -\log \phi(x_{t+1}; w_t, \sigma^2) < -\log \phi(x_{t+1}; x_{t+1}, \sigma^2) + \beta \\ \Rightarrow & \frac{1}{\sigma\sqrt{2\pi}} \exp \frac{-(x_{t+1} - w_t)^2}{2\sigma^2} > \frac{1}{\sigma\sqrt{2\pi}} e^{-\beta} \\ \Rightarrow & |x_{t+1} - w_t| < \sqrt{2\beta\sigma^2} \\ \Rightarrow & x_{t+1} \in (w_t - \sqrt{2\beta\sigma^2}; w_t + \sqrt{2\beta\sigma^2}). \end{aligned} \tag{S1.6}$$

By construction, the distribution of true segment data points $f(x_{t+1}|\delta_{t+1} = 0)$ is symmetric with mean w^*, and the same applies to the background point distribution. Thus, the overall distribution of the points used to update the w_t estimate is a truncation of a symmetric unimodal distribution. In the present case, it is a truncated normal with limits $(w_t - \sigma\sqrt{2\beta}, w_t + \sigma\sqrt{2\beta})$, based on (S1.6); more generally it is a truncated variant of the parent distribution with symmetric limits of the form $w_t \pm a$, and parent mean w^* (that the acceptance set is an interval follows from the unimodality of f).

This means that $f(x_{t+1}|\hat{\delta}_{t+1} = 1)$ satisfies the requirements for Lemma 1 with $\mu = w^*$, which implies the "convexity" condition (S1.2):

$$\inf_{(w-w^*)^2>\epsilon} (w - w^*)\mathbb{E}H(x_{t+1}, w) = \inf_{(w-w^*)^2>\epsilon} (w - w^*)(w - \mathbb{E}(x_{t+1}|\hat{\delta}_{t+1} = 1)) > 0$$

Following a similar approach – conditioning on δ_{t+1} – and using the law of total variance it can be shown that the variance of $\mathbb{E}(w - x_{t+1})$ is finite, as required for the condition (S1.4), and so $w_t \xrightarrow{a.s.} w^*$.

Remark. So far, we assumed that segment length $k = 1$. If segments occur and are tested in non-overlapping windows of any fixed size $k \geq 2$, the result is similar: $\forall j \in [t - k + 1; t], \hat{\delta}_{j+1} = 1$ if:

$$-\sum_{i=t-k+1}^{i=t} \log \phi(x_{i+1}; w_{t-k}, \sigma^2) < -\sum_{i=t-k+1}^{i=t} \log \phi(x_{i+1}; \bar{x}_k, \sigma^2) + \beta,$$

where $\bar{x}_k = \sum_{i=t-k+1}^{t} x_{i+1}/k$. This can be expressed as truncation limits for accepted $\bar{x}_k$, analogously to (S1.6):

$$\begin{aligned}
\beta &> -\sum_{i=t-k+1}^{t} \log \phi(x_{i+1}; w_{t-k}, \sigma^2) + \sum_{i=t-k+1}^{i=t} \log \phi(x_{i+1}; \bar{x}_k, \sigma^2) \\
&= \sum_{i=t-k+1}^{t} \frac{(x_{i+1} - w_{t-k})^2}{2\sigma^2} - \frac{(x_{i+1} - \bar{x}_k)^2}{2\sigma^2} \\
&= \frac{1}{2\sigma^2} \sum_{i=t-k+1}^{t} (w_{t-k} - \bar{x}_k)^2 \\
&\Rightarrow \sqrt{2\beta\sigma^2/k} > |w_{t-k} - \bar{x}_k|. \qquad \text{(S1.7)}
\end{aligned}$$

The pdf of $\bar{x}_k$ is a k-fold convolution of $f(x)$. Since f, as shown earlier, is symmetric unimodal, so is their convolution, and hence the distribution of $\bar{x}_k$, with a mean $\mu = w^*$ (Purkayastha, 1998). In the special case when f is the normal pdf, this can also be shown directly from Gaussian properties. Then Lemma 1 implies condition (S1.2), and the rest of the proof follows as before.

When all segment positions (overlapping or not) are tested, the background point acceptance rule is:

$$\hat{\delta}_t = 1 \text{ if } x_t \in \bigcap_{1\leq k\leq l} S_k, \text{ with } S_k = \{x_t : F(t-1)+C^0(x_t) < F(t-k)+C(x_{t-k+1:t})+\beta\}.$$

As demonstrated earlier, $S_1 = (w_t - \sqrt{2\beta\sigma^2}, w_t + \sqrt{2\beta\sigma^2})$. Define three sets of x_t based on which rules they pass: $X_1 = x_t \in S_1$, $X_a = x_t \in S_1 \cap S_{\geq 2}$, $X_r = x_t \in S_1 \setminus S_{\geq 2}$, and let P_a and P_r be the probabilities of the corresponding x_t sets. Clearly, $X_1 = X_a \cup X_r$. We are interested in the mean of the points accepted as background, i.e. $\mathbb{E}X_a$. Assume w.l.o.g. $\mu = 0, \sigma = 1, w_t > \mu$, as the other case is symmetric. We will now show that for sufficiently large n, $\mathbb{E}X_a < w_t$, satisfying (S1.2).

Using the conditional mean formula:

$$\begin{aligned} \mathbb{E}X_1 &= P_r\mathbb{E}X_r + P_a\mathbb{E}X_a \\ \mathbb{E}X_a &= \mathbb{E}X_1/(1-P_r) - P_r\mathbb{E}X_r/(1-P_r). \end{aligned} \tag{S1.8}$$

Assume for now $\mathbb{E}X_r = \mu = 0$. Then, to obtain $\mathbb{E}X_a < w_t$, we need:

$$\mathbb{E}X_1/w_t < 1 - P_r. \tag{S1.9}$$

Denote $p = \sqrt{2\beta}$, which is an increasing function of n, and consider the growth of both sides of (S1.9) as n increases. For the Gaussian or other distributions in the exponential family with mean μ, truncated to a symmetric region $(-a, a)$, it is known that $Var(X|S;\mu) = \frac{d}{d\mu}\mathbb{E}(X|S;\mu)$ (Zidek and van Eeden, 2003). Then (denoting $S_1' = (-p; +p)$):

$$\begin{aligned} \mathbb{E}X_1 &= w_t + \mathbb{E}(X|S_1'; -w_t) \\ &= w_t + \mathbb{E}(X|S_1'; 0) + \int_0^{w_t} \frac{d\mathbb{E}(X|S_1'; -a)}{da} da \\ &= w_t + 0 - \int_0^{w_t} \frac{d\mathbb{E}(X|S_1'; a)}{da} da \\ &< w_t - w_t \min_{0 \leq a \leq w_t} Var(X|S_1'; a). \end{aligned}$$

Hence:

$$\mathbb{E}X_1/w_t < 1 - Var(X|S_1'; w_t) = 1 - \int_{w_t-p}^{w_t+p} x^2 f(x) dx,$$

where $f(x)$ is the pdf of x_t given $x \in S_1$. Hence, this side grows with p as $-x^2 f(x)$.

To analyse P_r, we first simplify the background acceptance condition. For any $k \geq 2$, by definition of F and additivity of C, we have:

$$\begin{aligned} F(t-k) + C(x_{t-k+1:t}) + \beta &= F(t-k) + C(x_{t-k+1:t-1}; \bar{x}_k) + C(x_t; \bar{x}_k) + \beta \\ &= F(t-k) + C(x_{t-k+1:t-1}; \bar{x}_{-t}) + (k-1)d(\bar{x}_k, \bar{x}_{-t}) + d(\bar{x}_k, x_t) + \beta \\ &= F(t-1) + A(t-1) + (k-1)d(\bar{x}_k, \bar{x}_{-t}) + d(\bar{x}_k, x_t), \end{aligned}$$

where d is some distance function, $\bar{x}_{-t}$ is the mean of points $x_{t-k+1:t-1}$, and $A(t) \geq 0$ is a constant depending only on $x_{1:t}$.

It is also helpful to note that $F(t-1) \leq F(t-k) + C^0(x_{t-k+1:t-1})$, hence:

$$\begin{aligned} A(t-1) &= F(t-k) + C(x_{t-k+1:t-1}; \bar{x}_{-t}) + \beta - F(t-1) \\ &\geq C(x_{t-k+1:t-1}; \bar{x}_{-t}) + \beta - C^0(x_{t-k+1:t-1}) \\ &\geq \beta - (k-1)d(\bar{x}_{-t}, w_{t-1}). \end{aligned} \tag{S1.10}$$

This corresponds to the case when all $x_{t-k+1:t-1}$ were identified as background.

Using the Gaussian cost, i.e. $d(a,b) = (a-b)^2/2$, and recursive formula for the mean, the acceptance condition for k becomes:

$$\begin{aligned} &F(t-1) + C^0(x_t) < F(t-1) + A(t-1) + \frac{k-1}{2k^2}(x_t - \bar{x}_{-t})^2 + \frac{(k-1)^2}{2k^2}(x_t - \bar{x}_{-t})^2 \\ &\Rightarrow (x_t - w_{t-1})^2 < 2A(t-1) + \frac{k-1}{k}(x_t - \bar{x}_{-t})^2. \end{aligned} \tag{S1.11}$$

By substituting in the value of $A(t-1)$ from (S1.10), we obtain the following lower bound for $P(x \in S_k | x)$:

$$\begin{aligned} P(x \in S_k|x) &\geq P\left((x_t - w_{t-1})^2 < 2\beta - (k-1)(\bar{x}_{-t} - w_{t-1})^2 + \frac{k-1}{k}(x_t - \bar{x}_{-t})^2\right) \\ &= P\left((x_t - w_{t-1})^2 - \frac{k-1}{k}(x_t - \bar{x}_{-t})^2 + (k-1)(\bar{x}_{-t} - w_{t-1})^2 < 2\beta\right) \\ &\geq P\left((x_t - w_{t-1})^2 + (k-1)(\bar{x}_{-t} - w_{t-1})^2 < 2\beta\right) \\ &\geq 1 - \mathbb{E}\left((x_t - w_{t-1})^2) + (k-1)(\bar{x}_{-t} - w_{t-1})^2\right) / (2\beta) \\ P(x \notin S_k|x) &\leq \mathbb{E}\left((x_t - w_{t-1})^2) + (k-1)(\bar{x}_{-t} - w_{t-1})^2\right) / (2\beta)\,. \end{aligned}$$

Thus, we have the following bound for P_r at any k:

$$\begin{aligned} P_r &= \int_{w_t - p}^{w_t + p} P(x \notin S_k|x) f(x) dx \\ &\leq \int_{w_t - p}^{w_t + p} O(x^2/p^2) f(x) dx. \end{aligned} \tag{S1.12}$$

Therefore, as N increases, $1 - P_r$ grows faster than $1 - \mathbb{E}X_1/w_t = 1 - \int_{w_t-p}^{w_t+p} x^2 f(x)dx$. This means that $\exists p_0$, and thus $\exists n_0$, such that for $n > n_0$, and thus $p > p_0$, (S1.9) holds.

So far we assumed $\mathbb{E}X_r = \mu$. Clearly, for larger values of $\mathbb{E}X_r$, $\mathbb{E}X_a$ is even

smaller and (S1.2) is satisfied.

For the case when $\mathbb{E}X_r < \mu$, consider the worst case scenario $\mathbb{E}X_r = w_t - p$ (this is the bound to X_1, and thus to X_r, imposed by S_1). Similarly to (S1.8), we need:

$$\begin{aligned}
&\mathbb{E}X_a < w_t \\
\Rightarrow &\mathbb{E}X_1/(1-P_r) - P_r(w_t - p)/(1-P_r) < w_t \\
\Rightarrow &\mathbb{E}X_1/w_t - P_r + P_r p/w_t < 1 - P_r \\
\Rightarrow &P_r p/w_t < 1 - \mathbb{E}X_1/w_t \\
\Rightarrow &P_r p/w_t < \int_{w_t-p}^{w_t+p} x^2 f(x)dx.
\end{aligned}$$

However, based on (S1.12), $P_r p/w_t \leq \int_{w_t-p}^{w_t+p} O(x^2/p) f(x)dx$, so again $\exists n > n_0$ such that $\mathbb{E}X_a < w_t$, and condition (S1.2) holds.

And so overall $\mathbb{E}(x_t|\hat{\delta}_t = 1)$ satisfies condition (S1.2). Since the distribution of accepted x_t still has bounded support imposed by S_1, condition (S1.4) still holds, and the learning rate condition (S1.3) holds as before, implying convergence.

□

S1.2 When the global mean of segments does not match the background mean

Consider now that the overall mean of segment points $\mu_S = \int_{-\infty}^{\infty} x f_S(x)dx \neq w^*$, in particular $\mu_S > w^*$. Then if $w^* < w_t < \mu_S$, any segment points that were misclassified as background will (on average) push the estimates away from the background mean, in violation of the "convexity" condition (S1.2).

We assume that each segment point is followed by no less than n background points. Then, as $n \to \infty$, $w_t \xrightarrow{a.s} w^*$. For every finite n, $\exists \epsilon > 0$ such that $P(|w_t - w^*| > \epsilon) = 0$.

Proof. Suppose that a misclassification at time T is followed by n correctly classified background points: $\delta_T = 0$, $\delta_t = 1$ for $t \in [T+1; T+n]$, $\hat{\delta}_t = 1$ for $t \in [T; T+n]$. For the points $t \in [T+1; T+n]$, almost sure convergence of w_t was established above, i.e. for all $\epsilon > 0$, there exists a t_0 such that $\forall t : n \geq t \geq t_0, P(|w_{T+t} - w^*| < \epsilon) = 1$.

Therefore, given $n \geq t_0$:

$$
\begin{aligned}
& P(|w_{T+n} - w^*| < |w_{T-1} - w^*|) = 1 \\
\Rightarrow & \begin{cases} P(w_{T+n} - w_{T-1} < 0) = 1, \text{ if } w_{T-1} - w^* > 0 \\ P(w_{T+n} - w_{T-1} > 0) = 1, \text{ if } w_{T-1} - w^* < 0 \end{cases} \\
\Rightarrow & \inf_{w_{T-1} \neq w^*} (w_{T-1} - w^*) \mathbb{E}(w_{T+n} - w_{T-1}) < 0.
\end{aligned} \tag{S1.13}
$$

Indexing the segment-background cycles by i, denote the first estimate of that segment by w_i', so the set of these estimates are:

$$\{w_i'\} = \{w_1, \ldots, w_{T-2-n}, w_{T-1}, w_{T+n}, w_{T+1+2n}, \ldots\}.$$

The elements of this sequence can be expressed recursively as:

$$w_{i+1}' = w_i' - \gamma_i' H'(\{x_i'\}, w_i'),$$

with $\{x_i'\} = \{x_t : i(n+1) \leq t \leq i(n+1) + n\}$.

From (S1.13), $\mathbb{E}(w_i' - w_{i+1}') = \gamma_i' \mathbb{E} H'(\{x_i'\}, w_i')$ is "convex" as defined in (S1.2), and because $\gamma_i' > 0$ so is $\mathbb{E} H'(\{x_i'\}, w_i')$.

Let $\gamma_i' = \frac{1}{i(n+1)+1}$. Then:

$$
\begin{aligned}
H'(\{x_i'\}, w_i') &= \sum_{t=i(n+1)}^{i(n+1)+n} \gamma_t (w_{t-1} - x_t)/\gamma_i' \\
&= \sum_{t=i(n+1)}^{i(n+1)+n} \frac{i(n+1)+1}{t+1} (w_{t-1} - x_t) \\
&< (n+1)(w_{t-1} - x_t).
\end{aligned}
$$

So for $n < \infty$, conditions (S1.3)–(S1.4) are satisfied as well, and $w_i' \xrightarrow{a.s.} w^*$. (When $n \to \infty$, the convergence conditions are satisfied directly without using the sequence $\{w_i'\}$.) □

S1.3 Martingale Approach

We can also describe the update process over the background points using martingales. The algorithm estimates are random variables w_t; let $\{\mathcal{W}_t\}$ be the sequence of σ-algebras such that for each t, w_t is measurable with respect to $\mathcal{W}_t$. Using Lemma

1, and assuming $w^* < w_t$ again, within each cycle the estimates comprise a supermartingale $\mathbb{E}(w_{t+1}|\mathcal{W}_t) < w_t$ over the points $T \leq t < \min(T + n, FHT_w(w^*))$, here $FHT_x(a) = \inf\{t : x_t \leq a\}$ is the first hitting time of the process realisation $\{x_t\}$ to value a.

Consider again the problematic case when the global mean does not match the background mean and misclassification pushes the estimate away from the background mean, i.e. $w^* < w_{T-1} < w_T < \mu_S$. In order for w'_i to converge, we need the perturbed estimates to return to a value below w_{T-1} in each cycle. At the extremes, we have:

$$
\begin{aligned}
&FHT_w(w_T) = T && \text{starting position} \\
&FHT_w(w_{T-1}) < \infty && \text{for sufficiently large } n \text{, because } w_t \xrightarrow{a.s.} w^*.
\end{aligned}
$$

Clearly, $FHT_w(w_{T-1}) \leq FHT_w(w^*)$. However, the number of background points n required to satisfy $FHT_w(w_{T-1}) < T + n$ will depend on five factors: the distribution f_B and penalty p (since they determine the distribution of update values H), the size of estimated background set at time T (as it determines the relevant γ_t), and w_{T-1} and w_T.

In practice, n is bounded by the available data, so there is a non-zero probability that, over the segment-background cycles indexed by i:

$$\max_i FHT_w(w_{T_i-1}) > T_i + n.$$

In that case, define $b = \min\{a : \forall i, FHT_w(a) \leq T_i + n\}$; the final estimate of w'_i will be bound by $[w^*, b]$. As n increases, $P(|w^* - b| > \epsilon) \to 0$ for any $\epsilon > 0$.

Similar reasoning applies when $\mu_S < w_T < w_{T-1} < w^*$.

S2 Proof of Theorem 2 (Consistency)

Proof. Some general consistency results for changepoint detection by penalised cost methods are given in Fisch et al. (2018). In particular, an equivalent of our Theorem 2 is established for any algorithm that detects changepoints by exactly minimising a cost $F(n; \{s_i, e_i\}, \theta, \hat{\mu}, \hat{\sigma})$, where $\hat{\cdot}$ marks robust estimates of background parameters. While the original statement uses the median and interquartile range of $x_{0:n}$ for $\hat{\mu}$ and $\hat{\sigma}$, the proof only requires that the estimates satisfy certain upper bounds on deviations from the true values. Therefore, we will first show that the online estimates produced by Algorithm 1 are within these bounds, and then follow the

rest of the proof from Fisch et al. (2018).

Noting again that Algorithm 1 is effectively a stochastic gradient descent procedure, with each data point seen precisely once, we can use the error bound on estimates produced by such algorithms as provided in Theorem 7.5 of Harvey et al. (2019):

Theorem 1 ((Harvey et al., 2019)). *Let function $f(w)$ be 1-strongly convex and 1-Lipschitz. A stochastic gradient algorithm for minimising this function runs for T cycles, and at each cycle updates the estimate as in (S1.1) with $\gamma_t = 1/t$, $\mathbb{E}H = \nabla f(w)$. Then:*

$$P\left(\|w_t - w^*\|^2 \leq O\left(\frac{\log(1/\delta)}{t}\right)\right) \geq 1-\delta.$$

Using $\delta = n^{-\epsilon}$, and assuming without loss of generality that $\sigma_0 = 1$, we can establish an upper bound on the error of background parameters estimated by Algorithm 1 after n cycles:

$$P\left((\hat{\mu}-\mu_0)^2 \leq O\left(\frac{\log(n^\epsilon)}{n}\right)\right) = P\left(|\hat{\mu}-\mu_0| \leq O\left(\sqrt{\epsilon}\sqrt{\frac{\log n}{n}}\right)\right) \geq 1-n^{-\epsilon}$$

$$P\left(|\hat{\sigma}^2-\sigma_0^2| \leq O\left(\sqrt{\epsilon}\sqrt{\frac{\log n}{n}}\right)\right) \geq 1-n^{-\epsilon}.$$

Application of Boole's inequality leads to:

$$P\left(|\hat{\mu}-\mu_0| \leq D_1\sigma_0\sqrt{\frac{\log(n)}{n}}, \left|\frac{\hat{\sigma}^2}{\sigma_0^2}-1\right| \leq D_2\sqrt{\frac{\log(n)}{n}}\right) \geq 1-C_1 n^{-\epsilon}, \tag{S2.14}$$

for some constants C_1, D_1, D_2 and sufficiently large n. Since the objective function f in our algorithm is the Gaussian log-likelihood (i.e., the updates $\mathbb{E}H$ approximate its gradient), for any given segmentation it is 1-strongly convex. For other functions, overall consistency can still be achieved similarly, but the convergence rate may be slower than $n^{-\epsilon}$.

Having established the bound on estimate errors, we can use Lemma 9 from Fisch et al. (2018) and the proof method reported there.

First, introduce an event E based on a combination of bounds limiting the behaviour of Gaussian data $x_{1:n}$, which for any $\epsilon > 0$, occurs with probability $P(E) > 1 - C_2 n^{-\epsilon}$, with some constant C_2 and sufficiently large n (Lemmas 1 and 2 in Fisch et al. (2018)). Conditional on this event, the following lemma holds for the epidemic cost F defined as in the main text, eq. 5:

Lemma 2 ((Fisch et al., 2018))**.** *Let* $\{\tau\}$ *be the set of true segment positions* $\{(s_i, e_i)\}$*, and* θ *the vector of true segment means. Assume* E *holds, and some* $\hat{\mu}, \hat{\sigma}$ *are available for which the event in* (S2.14) *holds. Then, there exist constants* C_3 *and* n_1 *such that when* $n > n_1$*,*

$$F(n; \{\tau\}, \theta, \hat{\mu}, \hat{\sigma}) - F(n; \{\tau\}, \theta, \mu_0, \sigma_0) < C_4 \log n.$$

This lemma, together with results established for classical changepoint detection, can be used to show that the cost of any inconsistent solution will exceed the cost based on true segment positions and parameters (Proposition 8 in Fisch et al. (2018)):

Proposition 1 ((Fisch et al., 2018))**.** *Define* $\{\tau'\}$ *to be any set of segments* $\{(s_i, e_i)\}$ *that are not consistent (as defined in the main text, eq. 7). Let* $\tilde{\theta} = \operatorname{argmin}_\theta F(n; \theta)$ *be the parameters estimated by minimising the cost for a given segmentation (i.e. the vector of means and/or variances of* $x_{s_i:e_i}$ *for each* i*). Assume* E *holds. Then there exist constants* C_4 *and* n_2 *such that, when* $n > n_2$*:*

$$F(n; \{\tau'\}, \tilde{\theta}, \hat{\mu}, \hat{\sigma}) \geq F(n; \{\tau\}, \theta, \hat{\mu}, \hat{\sigma}) + C_3 \log(n)^{1+\delta/2}.$$

See the original publication for a detailed proof of these results.

Finally, for a given set of changepoints, using fitted maximum-likelihood parameters by definition results in minimal cost:

$$F(n; \{\tau\}, \theta, \hat{\mu}, \hat{\sigma}) \geq F(n; \{\tau\}, \tilde{\theta}, \hat{\mu}, \hat{\sigma}).$$

Thus, when Proposition 1 holds, we have:

$$F(n; \{\tau'\}, \tilde{\theta}, \hat{\mu}, \hat{\sigma}) > F(n; \{\tau\}, \tilde{\theta}, \hat{\mu}, \hat{\sigma}),$$

and an exact minimisation algorithm will always find a solution in the consistent set. The overall probability of the events required for Proposition 1 is a combination of $P(E)$, established before, and (S2.14), which by Boole's inequality is:

$$P > 1 - C_5 n^{-\epsilon},$$

for any $\epsilon > 0$, $n > n_3$ and some constants n_3, C_5. □

S3 Pruning of Algorithm 1

Much of the improvement in performance of change estimation algorithms comes from pruning of the search space. So far, the cost function was only required to be block-additive (see (2.2) in the maintext). Most lihelihood-based costs are also superadditive over points: for all $a \leq b < c$,

$$C(x_{a:c}) \geq C(x_{a:b}) + C(x_{b+1:c}). \tag{S3.15}$$

In that case, standard pruning (Killick et al., 2012) can be applied to Algorithm 1, and the resulting solution will still be optimal, as shown in Proposition 2.

To implement this, the search set for F_S in step 4 would be changed to:

$$F_S = \min_{t' \in K}(F(t') + C(x_{t'+1:t}) + \beta),$$

with K initialized to $\{0\}$ and updated with a new step 11a:

$$\text{11a. } K = K \cap \{s : F(s) + C(x_{s+1:t}) < F(t),\ t + 1 - l \leq s < t\} \cup \{t\}.$$

Proposition 2. *Assume a cost function C such that (S3.15) applies. If, for some $s > t - l$:*

$$F(s) + C(x_{s+1:t}) \geq F(t),$$

then $s + 1$ will not be a segment start point in the optimal solution, and can be excluded from consideration for all subsequent $t' > t$.

Proof. For all $t' \leq s + l$, the proof applies as for other pruned algorithms (Killick et al., 2012):

$$F(t) + C(x_{t+1:t'}) + \beta \leq F(s) + C(x_{s+1:t}) + C(x_{t+1:t'}) + \beta \leq F(s) + C(x_{s+1:t'}) + \beta.$$

For $t' > s + l$, segment $(s + 1, t')$ will exceed the length constraint and thus cannot be part of the final segmentation. □

S4 Proof of Proposition 1 (Pruning of Algorithm 2)

Proof. Denote the true start and end of a nuisance segment as s_j, e_j. Consider the case when $s_j \in (t - A(n); t]$. Pruning at time t will not remove this point (i.e.

$s_j \notin \boldsymbol{k}_{pr,t}$) iff:

$$C^0(x_{t-A(n):s_j-1}) + C^N(x_{s_j:t}) < C^0(x_{t-A(n):m-1}) + C^N(x_{m:t}) + \alpha \log(n)^{1+\delta}$$

with m such that the right hand side is minimised and $m \neq s_j$.

Denote by $C(x_{a:b}; \hat{\mu}, \hat{\sigma})$ the Gaussian cost calculated with MLE estimates of the parameters (i.e. mean and variance of $x_{a:b}$). Note that since $C^0(x_{a:b}) = C(x_{a:b}; \mu_0, \sigma_0)$ and $C^N(x_{a:b}) = C(x_{a:b}; \hat{\mu}, \sigma_N)$, the required event to preserve s_j can be stated as

$$C(x_{t-A(n):s_j-1}; \mu_0, \sigma_0) + C(x_{s_j:t}; \hat{\mu}, \sigma_N) - \\ C(x_{t-A(n):m-1}; \mu_0, \sigma_0) - C(x_{m:t}; \hat{\mu}, \sigma_N) < \alpha \log(n)^{1+\delta}$$

We can establish the probability of this using the following bound (Proposition 4 in Fisch et al. (2018)):

Lemma 3. *Let $x_{1:n}$ be piecewise-Gaussian data. Choose any subset $x_{i:j}, 1 \leq i < j \leq n$, with a true changepoint at s, i.e., we have $x_t \sim \mathcal{N}(\mu_1, \sigma_1)$ for $t \in [i; s-1]$, and $x_t \sim \mathcal{N}(\mu_2, \sigma_2)$ for $t \in [s; j]$. Then, for any candidate changepoint τ and any $\epsilon > 0$, there exist constants B, n_0, K_1 such that:*

$$C(x_{i:s-1}; \mu_1, \sigma_1) + C(x_{s:j}; \mu_2, \sigma_2) - C(x_{i:\tau-1}; \hat{\mu}, \hat{\sigma}) - C(x_{\tau:j}; \hat{\mu}, \hat{\sigma}) \leq K_1 \log(n)$$

is true for all i, j with $P \geq 1 - Bn^{-\epsilon}$ when $n > n_0$.

Now take $i = s_j - A(n) + 1, j = s_j + A(n) - 1$. Note that there is one and only one changepoint within $x_{i:j}$ because of the required distance between changepoints. Applying Lemma 3 to such $x_{i:j}$ states that, conditional on an event with probability $P \geq 1 - Bn^{-\epsilon}$, the following is true for all $t \in [s_j, s_j + A(n))$:

$$\begin{aligned} &C(x_{t-A(n):s_j-1}; \mu_0, \sigma_0) + C(x_{s_j:t}; \hat{\mu}, \sigma_N) - C(x_{t-A(n):m-1}; \mu_0, \sigma_0) - C(x_{m:t}; \hat{\mu}, \sigma_N) \\ &\leq C(x_{t-A(n):s_j-1}; \mu_0, \sigma_0) + C(x_{s_j:t}; \mu_N, \sigma_N) - C(x_{t-A(n):m-1}; \hat{\mu}, \hat{\sigma}) - C(x_{m:t}; \hat{\mu}, \hat{\sigma}) \\ &\leq K_1 \log(n) < \alpha \log(n)^{1+\delta}, \end{aligned}$$

where we also used the fact that $\hat{\mu}, \hat{\sigma} = \operatorname{argmin}_{\mu,\sigma} C(x; \mu, \sigma)$.

Therefore, with the same probability, $s_j \notin \bigcup_{t=s_j}^{s_j+A(n)-1} \boldsymbol{k}_{pr,t}$. Also, $s_j \notin \bigcup_{t=s_j+A(n)}^{n} \boldsymbol{k}_{pr,t}$ because then $s_j \leq t - A(n)$ and is not considered in the pruning scheme, and clearly $s_j \notin \bigcup_{t=1}^{s_j-1} \boldsymbol{k}_{pr,t}$. The case for e_j follows by symmetry, and obviously no true changepoint can be pruned out if $s_j, e_j = \emptyset$, so the overall probability of retaining a true changepoint remains at $P \geq 1 - Bn^{-\epsilon}$. □

S5 Details of experiments

S5.1 ChIP-seq data analysis

Two ChIP-seq datasets of histone modifications in human immune cells were used in this section.

Broad Institute H3K27ac data was obtained from UCSC Genome Browser, GEO accession GSM733771, as mean read coverage in non-overlapping windows of 25 bp. We also retrieved an input control track for the same cell line from UCSC (GEO accession GSM733742). We analysed a window near the centromere of chromosome 1, between 120,100,000 to 120,700,000 bp. To improve runtime, data was downsampled to approximately 1000 points, each corresponding to mean coverage in a window of 500 bp.

UCI/McGill dataset was obtained from https://archive.ics.uci.edu/ml/datasets/chipseq. Mean read coverage at 1 bp resolution is provided, as well as peak annotations based on visual inspection. From this data, we used the H3K36me3 modification in monocyte sample ID McGill0104, AM annotation. Around the labels $L = \{(s_i, e_i)\}$ in each chromosome, we extracted read coverage for the window between $s - (e - s)$ and $e + (e - s)$ bp, $s = \min s_i$, $e = \max e_i$, and downsampled to about 1000 points as before. Based on visual inspection of the coverage, we chose a group of labels in chromosome 12, which provides a variety of annotations and a structure that appears to contain both nuisance shifts and signal peaks.

The datasets were analysed by the method proposed here (Algorithm 2), an epidemic detector from package **anomaly**, and the non-epidemic detector **not**. The length of the signal segments was limited to 50 kbp in **anomaly** and the proposed method. Global μ_0 was estimated as the median of the unsegmented data, and σ_0 as the median absolute deviation of the first differences of the data. We also used GFPOP, implemented in R package **PeakSegDisk** (Hocking et al., 2018): this detector has been developed specifically for ChIP-seq data processing, and models a change in Poisson rate parameter. For all methods, only segments with estimated $\theta > \mu_0$ are considered as detections, as we are a priori interested in regions of increased binding.

The detection thresholds (penalties) were calibrated by a simple grid search: all methods were first applied to the Broad Institute control track, with several possible penalty values. For each method, the value that produced three segments (including nuisances) on this data was then set as the penalty. These penalties were used directly in the Broad data analysis, and multiplied by 2 for analysing the UCI data

as the changes are much stronger in that dataset.

To evaluate the results quantitatively, we calculated the SIC based on each method's segmentation. We used Gaussian likelihood with parameters matching the detection status (i.e., estimated mean for the points in each segment, and $\hat{\mu}_0$ for points outside segments). The number of parameters was set to $3k + 2$: a mean and two endpoints for each of the k segments reported, and two for the background parameters.

S5.2 European mortality data analysis

We analysed Eurostat data of weekly deaths in Spain over a three year period between 2017 and 2020. Data was retrieved from `https://ec.europa.eu` Data Explorer, table `demo_r_mwk_05`. We use the 60-64 years age group in which these trends are visually clear and thus provide a ground truth.

Four methods were used to analyse this data. For the **proposed** and **anomaly** methods, we used the median and median absolute deviation of the first 52 weeks of the dataset as estimates of μ_0 and σ_0, respectively. Penalties in both were set to $3\log(n)^{1.1}$, as previously. The maximum length of signal segments was set to 10 weeks, to separate seasonal effects. In addition, **not** was used with default parameters, defining signal as regions where the mean exceeds $\mu_0 + \sigma_0$, and **PeakSegDisk** with a basic grid search to select the penalty that produces between 2–5 detections.

S6 Supplementary Tables

Table ST1: Consistency of the number and position of estimated changepoints when using Algorithm 1 with or without step 13 (online version of the algorithm). Time series were simulated in three different scenarios in 500 replications for each n. The mean number of reported segments and the TPR (fraction of replications when a changepoint was detected within $0.05n$ of each true changepoint) are shown. The number of true segments was 1, 3, and 1 for scenarios **one segment**, **multiple**, **heavy tail** respectively.

		(Full algorithm)		(without step 13)	
Scenario	n	Mean # segm.	TPR	Mean # segm.	TPR
**one segm.**	30	1.116	0.916	1.138	0.902
	90	1.060	0.998	1.082	0.998
	180	1.044	0.996	1.056	0.996
	440	1.030	0.994	1.034	0.994
	750	1.014	0.998	1.012	0.998
**multiple**	30	1.273	0.000	1.274	0.000
	90	1.374	0.010	1.376	0.008
	180	1.863	0.128	1.824	0.108
	440	2.894	0.814	2.862	0.776
	750	3.022	0.982	3.020	0.982
**heavy tail**	30	1.242	0.124	1.249	0.118
	90	1.485	0.594	1.496	0.580
	180	1.868	0.860	1.882	0.864
	440	2.832	0.984	2.836	0.980
	750	3.864	1.000	3.868	1.000

Table ST2: Final cost corresponding to segmentations obtained by Algorithm 1 (either full or excluding step 13), or to the true segmentation. Cost is the log-likelihood of a Gaussian epidemic changepoint model with the corresponding segmentation. Given numbers are mean ± SD over 500 iterations for each setting.

Scenario	n	Full algorithm	Without step 13	True
**one segm.**	30	53.1 ± 5.11	53.3 ± 5.35	52.8 ± 3.82
	90	141.3 ± 7.23	141.6 ± 7.58	141.2 ± 6.57
	180	271.2 ± 9.66	271.3 ± 9.86	271.2 ± 9.21
	440	644.6 ± 14.88	644.6 ± 14.87	644.6 ± 14.55
	750	1085.3 ± 20.68	1085.3 ± 20.6	1085.5 ± 20.55
**multiple**	30	48.8 ± 7.47	49.1 ± 7.64	74.2 ± 3.67
	90	145.5 ± 10.28	145.4 ± 10.39	170.4 ± 6.36
	180	286.1 ± 12.66	285.8 ± 12.56	304.5 ± 8.90
	440	677.4 ± 14.87	677.2 ± 14.98	683.8 ± 14.85
	750	1123.3 ± 17.55	1123.3 ± 17.56	1128.6 ± 17.51
**heavy tail**	30	68.6 ± 10.23	68.7 ± 10.32	68.2 ± 12.19
	90	198.7 ± 24.73	198.8 ± 24.79	190.2 ± 29.78
	180	389.8 ± 38.68	389.9 ± 38.89	371.9 ± 71.27
	440	951.0 ± 64.27	951.1 ± 64.35	889.7 ± 164.13
	750	1626.5 ± 87.24	1626.5 ± 87.00	1507.5 ± 324.36

Table ST3: The true positive rate of changepoint estimation by the proposed Algorithm 2 and alternative detectors. Data simulated in 1000 replications. The true positive rate is the fraction of iterations when a (signal) changepoint was detected within $0.05n$ of each true signal changepoint.

Scenario	n	Proposed	anomaly	aPELT	sparse	not
1	30	0.431	0.381	0.586	0.184	0.428
	60	0.712	0.508	0.814	0.07	0.692
	150	0.986	0.986	0.993	0.05	0.991
	220	0.996	0.999	0.998	0.021	1.000
2	30	0.639	0.92	0.853	0.946	0.055
	60	0.905	0.986	0.981	0.985	0.879
	150	0.994	1.000	1.000	1.000	1.000
	220	0.996	1.000	1.000	1.000	1.000
3	30	0.000	0.000	0.000	0.000	0.000
	60	0.001	0.003	0.001	0.000	0.000
	150	0.015	0.022	0.013	0.003	0.005
	220	0.041	0.047	0.037	0.01	0.014

Table ST4: Mean absolute errors of changepoint localization by the proposed Algorithm 2 and alternative detectors. Data simulated in 1000 replications. The localization error is defined as the number of data points between an estimated (signal) changepoint and the nearest true changepoint. These errors were averaged over all changepoints that the methods reported within $0.05n$ of a true changepoint.

Scenario	n	Proposed	anomaly	aPELT	sparse	not
1	30	0.248	0.284	0.270	0.365	0.278
	60	0.409	0.440	0.395	0.762	0.407
	150	0.640	0.696	0.604	2.94	0.681
	220	0.657	0.732	0.660	4.35	0.697
2	30	0.110	0.122	0.128	0.124	0.117
	60	0.174	0.181	0.184	0.182	0.202
	150	0.203	0.203	0.205	0.204	0.275
	220	0.187	0.187	0.182	0.184	0.235
3	30	0.233	0.115	0.141	0.122	0.504
	60	0.246	0.208	0.204	0.224	0.233
	150	0.823	0.430	0.447	0.458	0.609
	220	0.678	0.570	0.577	0.598	0.498

Table ST5: Performance of changepoint estimation by the proposed Algorithm 2 and alternative detectors with different segment length bounds l. Data simulated following scenario 1 (1 signal and 1 nuisance segment), with $n = 60$, in 500 replications. Metrics are mean number of segments (split by type for the proposed method), and true positive rate – fraction of iterations when a (signal) changepoint was detected within $0.05n$ of each true signal changepoint.

	method	$l = 0.25n$	$l = 0.33n$	$l = 0.4n$
mean # seg.	proposed (signal)	1.05	1.12	1.14
	proposed (nuis.)	0.98	0.92	0.89
	anomaly	2.71	2.51	2.45
	aPELT	3.14	2.96	2.96
	sparse	2.68	2.32	1.73
TPR	proposed	0.712	0.698	0.708
	anomaly	0.740	0.498	0.510
	aPELT	0.860	0.808	0.796
	sparse	0.538	0.068	0.030

References

Bottou, L. (1998) Online algorithms and stochastic approximations. In *Online Learning and Neural Networks* (ed. D. Saad). Cambridge, UK: Cambridge University Press.

Fisch, A. T. M., Eckley, I. A. and Fearnhead, P. (2018) A linear time method for the detection of point and collective anomalies. *arXiv preprint*.

Harvey, N. J. A., Liaw, C., Plan, Y. and Randhawa, S. (2019) Tight analyses for non-smooth stochastic gradient descent. In *Proceedings of the Thirty-Second Conference on Learning Theory* (eds. A. Beygelzimer and D. Hsu), vol. 99 of *Proceedings of Machine Learning Research*, 1579–1613. Phoenix, USA: PMLR.

Hocking, T. D., Rigaill, G., Fearnhead, P. and Bourque, G. (2018) Generalized functional pruning optimal partitioning (gfpop) for constrained changepoint detection in genomic data. *arXiv preprint*.

Killick, R., Fearnhead, P. and Eckley, I. A. (2012) Optimal detection of changepoints with a linear computational cost. *Journal of the American Statistical Association*, **107**, 1590–1598.

Purkayastha, S. (1998) Simple proofs of two results on convolutions of unimodal distributions. *Statistics & Probability Letters*, **39**, 97–100.

Zidek, J. V. and van Eeden, C. (2003) *Uncertainty, entropy, variance and the effect of partial information*, vol. 42 of *Lecture Notes–Monograph Series*, 155–167. Institute of Mathematical Statistics.
